# Host Suitability of House Fly, *Musca domestica* (Diptera: Muscidae), Pupae Killed by High or Low Temperature Treatment for a Parastoid, *Spalangia endius* (Hymenoptera: Pteromalidae)

**DOI:** 10.1100/2012/214907

**Published:** 2012-09-25

**Authors:** Kohei Ogawa, Katsura Ito, Tatsuya Fukuda, Shin-ichi Tebayashi, Ryo Arakawa

**Affiliations:** Faculty of Agriculture, Kochi University, 200 Monobe-otsu, Nankoku, Kochi 783-8502, Japan

## Abstract

The objective of this study was to establish a high quality progeny production system for the house fly parasitoid, *Spalangia endius* (Hymenoptera: Pteromalidae), by stockpiling hosts. We performed two host killing methods before host storage: (i) heat-killed by 30 min exposure to 50°C or (ii) freeze-killed by 10 min exposure to −80°C. The average number of parasitoids that emerged from nonstored house fly pupae after heat- or freeze-killing was not significantly different from live pupae. When house fly pupae stored at −20°C after heat-killing were supplied to *S. endius*, progeny production was significantly less than live pupae. Moreover, productivity became very low when house fly pupae refrigerated at 3°C after heat- or freeze-killing were supplied to *S. endius*. On the other hand, when house fly pupae stored at −80°C for 1 year after heat-killing were supplied to *S. endius*, the average number of parasitoids that emerged was not significantly different from live pupae. The average number of parasitoids that emerged from freeze-killed hosts kept for more than 8 weeks at −80°C was significantly fewer than live pupae. Thus, this study clarified that a higher-quality host can be maintained not only by simply storing at –80°C but also by adding heat treatment before storage.

## 1. Introduction

The house fly, *Musca domestica* L. (Diptera: Muscidae), is one of the most common pests of confined livestock such as dairy and poultry. The number of livestock farmers using biological control methods to control such pests is increasing because of the development of insecticide resistance and the general movement toward sustainable pest control systems including integrated pest management (IPM) [1]. In several livestock facilities, it was reported that the release of pteromalid parasitoids belonging to the genus *Spalangia,* together with other components, was useful in controlling fly populations at acceptable levels [2–5].

To use parasitoids for sustainable management, a mass rearing system of both parasitoids and hosts should be established. Although it is necessary to produce large numbers of hosts and parasitoids simultaneously and continuously, this demands a great deal of time and effort. In addition, commercial insectaries that produce fly parasitoids face a dilemma when customer demand for their products is low during the off-season from winter to early spring, and production is curtailed as a cost-saving measure [6]. The ability to stockpile high-quality hosts during the off-season would provide a way of scaling up parasitoid production more rapidly as the fly season approaches and would give producers a way to respond to fluctuations in demand for parasitoids ([7]; referred to in [6]).

Floate [8] clarified that hosts stored at −20°C in the refrigerator for at least 6 months are suitable for the production of pteromalid house fly parasitoids belonging to *Muscidifurax*. Geden and Kaufman [6] reported that production of *S. cameroni* on freeze-killed host pupae stored at 4°C for 2–8 weeks was 73%–78% compared with using live pupae. Furthermore, production of *S. cameroni* on heat-killed pupae stored at 4°C for 2 and 4 months was 83 and 64%, respectively, compared with using live pupae. Therefore, stockpiling killed fly hosts may be possible as adequate hosts of house fly parasitoids. Although the release of parasitized hosts is common in biological control of house flies by parasitoids, if there are unparasitized hosts, adult flies will emerge from those hosts. Therefore, the hosts should be pretreated to avoid adult emergence. Establishing long term storage of those hosts is effective in the mass rearing and mass releasing of parasitoids as biological control agents. The objective of this study was to clarify host suitability for long term storage of *S. endius*, which is a worldwide-distributed solitary parasitoid of at least 50 different host species in 9 families of Diptera [9], at low or high temperatures after heat- or freeze-killing.

## 2. Materials and Methods

### 2.1. Determination of Lethal Levels of Heat or Cold Treatment for House Fly Pupae as Hosts

House fly adults were collected from the livestock house of Kochi Agricultural High School (33°34′N, 133°39′E) and the Faculty of Agriculture, Kochi University (33°32′N, 133°40′E), Nankoku, Kochi, Japan in 2006. The flies were maintained with deionized water, sugar and skim milk in a net cage (290 mm × 290 mm × 290 mm) under room temperature conditions. As an ovipositional and breeding site, a glass cylinder with a medium culture of wheat bran 50 g, powder diet 50 g, fish flour 2.5 g, dry yeast 0.5 g, and deionized water 100 mL was supplied in the cage. When most eggs hatched into larvae, the cylinder was transferred to an incubator set at 25°C, 75% RH and a 16 h light and 8 h dark photoperiodic cycle (16L8D) and kept until pupation. Extracted fly pupae just after pupation were provided every 4 days for parasitoid oviposition. *Spalangia endius* was provided by Sumika Technoservice Corporation, Takarazuka, Hyogo, Japan. Adult parasitoids were held in clear plastic cups (25°C, 75% RH) with a drop of honey and a photoperiod of 16L8D. Parasitized hosts were held in a 25°C, 75% RH incubator until flies or parasitoids emerged. Treatments were conducted to determine lethal doses of heat and cold causing 100% mortality of fly pupae. For heat tests, 100 2-day-old pupae on an aluminum plate were placed in a drying sterilizer adjusted to 45, 50, 55, and 60°C for varying amounts of time ranging from 15 to 60 min. The treated hosts (three replicates of 100 for each temperature and exposure time) were transferred to an incubator maintained at 25°C, 60% RH, and 16L8D for fly emergence. For cold tests, groups of 2-day-old pupae (three replicates of 100 per treatment) were placed in an ultra-low freezer set at −80°C for 1, 3, 5, 7, or 10 min according to the method by Geden and Kaufman [6]. After that, they were placed under standard rearing conditions as before.

### 2.2. Effects of Host Storage Duration against Parasitoid Development

Heat- and freeze-killed hosts by the method mentioned above were kept in a refrigerator or freezer adjusted to 3, −20, or −80°C. Heat-killed hosts were kept for 8 and 12 weeks at 3°C, for 1, 4, 8, and 12 weeks at −20°C, and for 1, 4, 8, and 12 weeks and 1 year at −80°C. Freeze-killed hosts were kept for 1, 4, 8 and 12 weeks at 3°C, and for 1, 4, 8, 12 weeks and 1 year at −80°C.

The hosts stored in the same condition were divided into 10 or 15 replications of 20 pupae in a petri dish (55 mm diameter, 15 mm height). After resting for 3 hours at room temperature, one female *S. endius* within 7 days after emergence was released in each petri dish and held at 25°C, 60 RH, and 16L8D for 24 hours. At this time, a drop of honey was provided as food for the parasitoid inside the petri dish. After the parasitoid was taken out of the petri dish, hosts were maintained until parasitoids emerged under the same condition. The number of parasitoids that emerged was then recorded. The host pupae from which no parasitoids emerged after more than 30 days of being supplied to the parasitoid were dissected to determine whether they had been parasitized or not. When a dead body of an adult parasitoid was found in a host, it was considered as an emerged individual. For comparison, the same experiments were conducted on 2-day-old live pupae that had not been stored or just after being heat-killed.

### 2.3. Statistical Analysis

Data analysis in this study was performed with SPSS Statistics Software version 19. One-way ANOVA followed by Tukey's HSD test was used for comparing differences between multiple groups. Differences were considered significant at *P* < 0.05.

## 3. Results

### 3.1. Determination of Lethal Levels of Heat and Cold for Host Pupae

Exposure of house fly pupae to 45°C for 15–45 min and to 50°C for 15 min resulted in <100% mortality and other treatment induced 100% mortality (Table 1). Exposure of pupae to −80°C for ≧3 min resulted in >90% mortality and exposure times of 10 min were needed for 100% mortality (Table 2). In this study, we selected two host killing treatments for examination: (i) heat-killed hosts, 30 min exposure to 50°C and (ii) freeze-killed hosts, 10 min exposure to −80°C.

### 3.2. Effect of Host Storage on Parasitoid Development

The average number of parasitoids that emerged from hosts stored for 8 or 12 weeks at 3°C after being heat-killed was 2.9 and 0.4, respectively. These numbers were significantly different from control (Table 3).

The average number of parasitoids that emerged from heat-killed hosts stored for 12 weeks at −20°C was 3.1. This number was significantly different from live hosts and non-stored, heat killed hosts (Table 3). When heat-killed hosts stored for ≦8 weeks at −20°C were supplied to parasitoids, the average number of parasitoids that emerged was not significantly different from non-stored, heat-killed hosts but was significantly different from live hosts. The average number of parasitoids that emerged from heat-killed hosts stored for 1, 4, 8, 12 weeks and 1 year at −80°C was not significantly different from live hosts and non-stored, heat-killed hosts (Table 3).

The average number of parasitoids that emerged from 10 freeze-killed hosts stored for 1 or 4 weeks at 3°C was significantly different from live hosts and non-stored, freeze-killed hosts (Table 4). None of parasitoids emerged from freeze-killed hosts were stored for 8 or 12 weeks at 3°C.

The average number of parasitoids that emerged from freeze-killed hosts stored for 1 or 4 weeks at −80°C was not significantly different from live hosts, although the average number of parasitoids that emerged from freeze-killed hosts stored for 8, 12 weeks and 1 year was significantly different from live hosts (Table 4). The average number of parasitoids that emerged from stored, freeze-killed hosts, with the exception of 12 weeks, was not significantly different from non-stored, freeze-killed hosts.

## 4. Discussion

Geden and Kaufman [6] summarized that when house fly pupae were used as the hosts of parasitoids, killed pupae had 3 advantages over live pupae. First, in the field surveys of parasitoid activity using laboratory reared pupae as sentinel hosts, live pupae are only useful for a few days after pupation. In contrast, killed hosts for field surveys are possible to place for a long time. Second, killed pupae can also be used in foreign exploration efforts to establish colonies of introduced biological control agents in areas where live hosts are not available. Third, as killed hosts block emerging adult flies from pupae that escape parasitism, placed killed hosts in livestock do not release live house fly. Thus, when parasitoids grow in house fly pupae, high-quality killed hosts have a higher applicability than live hosts.

For the house fly parasitoids, *M. raptor*, *M. raptorellus,* and *M. zaraptor*, freeze-killed hosts clearly showed effective productivity [8, 10]. But for *S. cameroni* and *S. endius*, it was reported that freeze-killed hosts were not effective [8, 11]. Geden and Kaufman [6] indicated that freeze-killed house fly pupae (10 min at −80°C) produced about 80% as many *S. cameroni* progeny as live hosts, and in the case of heat-killed pupae (30 min at 50°C) production was not significantly different from live pupae. On the other hand, in this study we showed that production of *S. endius* from heat- or freeze-killed pupae was not significantly different from live pupae (Tables 3 and 4). In the genus *Spalangia*, it is necessary to investigate the effectiveness of host preservation by using optimal age and suitable temperature treatment. From these results, we believe that killed house fly pupae subjected to heat or cold treatment are sufficiently applicable to parasitoids for rearing.

Progeny production by *S. cameroni* on freeze-killed pupae stored for more than 6 weeks and heat-killed pupae stored for more than 3 months was significantly less than the production from live pupae refrigerated at 4°C after killing [6]. In this study, when *S. endius* was offered pupae stored at −20°C after being heat-killed, progeny production was significantly less than for live pupae. Moreover, productivity became very low when house fly pupae, refrigerated at 3°C after heat or cold treatment, were supplied to *S. endius* (Tables 3 and 4). However, when *S. endius* was offered heat-killed pupae stored at −80°C for 1 year, the average number of progeny was not significantly different from live pupae. The average number of parasitoids that emerged from freeze-killed hosts kept for more than 8 weeks at −80°C was significantly fewer than live pupae. Thus, the quality of stockpiled hosts killed by heat or freeze treatment can be maintained by storing them at a lower temperature. With respect to degradation of the quality of stored, freeze-killed house fly hosts, Petersen and Matthews [10] reported that reduced suitability of freeze-killed hosts on the reproduction of *M. zaraptor* was due to desiccation. In contrast, Kaufman and Geden [1] suggested that freezing treatment resulted in damage to the house fly pupae and made them more vulnerable to colonization by decomposing and saprophytic microorganisms. In our study, although it was not determined why the progeny on killed hosts decreased compared to live hosts, the desiccation of those hosts may not induce the emergence of parasitoids as was the case for Peterson and Matthews [10]. This study clarified that higher-quality hosts can be maintained not only by simply storing at−80°C but adding heat treatment before storage (Tables 3 and 4). Storage at–80°C after heat treatment reduces desiccation by thermal denaturation of the surface of the fly pupa and might suppress the activity of decomposing and saprophytic microorganisms. The reproduction ability of parasitoids that emerged from heat-killed hosts may be clarified in a subsequent paper.

## Figures and Tables

**Table 1:** Mean ± SE house fly mortality (%) after heat treatment for determination of lethal level of heat.

Temperature (°C)	Time (min)			
	15	30	45	60
45	4.7 ± 0.9	54.7 ± 2.9	98.6 ± 0.9	100 ± 0
50	89.7 ± 1.2	100 ± 0	100 ± 0	100 ± 0
55	100 ± 0	100 ± 0	100 ± 0	100 ± 0
60	100 ± 0	100 ± 0	100 ± 0	100 ± 0

**Table 2:** Mean ± SE house fly mortality (%) after cold treatment for determination of lethal level of cold.

Exposure time at −80°C (min)	House fly pupal mortality
0 (controls)	1.3 ± 0.9
1	13.0 ± 4.0
3	91.0 ± 4.6
5	99.0 ± 0.6
7	98.3 ± 0.3
10	100 ± 0

**Table 3:** Progeny production of *S. endius* on house fly pupae treated with different temperatures and storage periods after heat treatment.

Storage temperature (°C)	N*	Storage period (weeks)	Pupal treatment	No. progeny produced (mean±SE)	
—	14	—	Live	13.5 ± 1.0	a**
—	10	—	Heat-killed	9.4 ± 0.9	ab
3	10	8	Heat-killed	2.9 ± 0.9	cd
3	10	12	Heat-killed	0.4 ± 0.2	d
−20	10	1	Heat-killed	5.7 ± 1.2	bcd
−20	10	4	Heat-killed	6.7 ± 0.6	bc
−20	10	8	Heat-killed	6.6 ± 1.2	bc
−20	10	12	Heat-killed	3.1 ± 0.6	cd
−80	9	1	Heat-killed	10.3 ± 1.7	ab
−80	10	4	Heat-killed	8.1 ± 2.0	abc
−80	10	8	Heat-killed	8.0 ± 1.8	abc
−80	10	12	Heat-killed	10.5 ± 1.3	ab
−80	15	52	Heat-killed	10.5 ± 1.2	ab

*N is indicated replications.

**Means within column followed by the same letter are not significantly different at *P* = 0.05 (Tukey's HSD test).

**Table 4:** Progeny production of *S. endius* on house fly pupae treated with different temperatures and storage periods after cold treatment.

Storage temperature (°C)	N*	Storage period (weeks)	Pupal treatment	No. progeny produced (mean±SE)	
—	14	—	Live	13.5 ± 1.0	a**
—	9	—	freeze-killed	10.0 ± 0.5	ab
3	10	1	freeze-killed	1.0 ± 0.4	de
3	10	4	freeze-killed	0.1 ± 0.1	e
3	10	8	freeze-killed	0	
3	10	12	freeze-killed	0	
−80	9	1	freeze-killed	10.0 ± 1.8	ab
−80	10	4	freeze-killed	10.2 ± 0.9	ab
−80	10	8	freeze-killed	7.1 ± 1.3	bc
−80	10	12	freeze-killed	4.1 ± 0.8	cd
−80	15	52	freeze-killed	7.3 ± 0.9	bc

∗*N* is indicated replications.

**Means within column followed by the same letter are not significantly different at *P* = 0.05 (Tukey's HSD test).
